# *Lactiplantibacillus plantarum* Strains Modulate Intestinal Innate Immune Response and Increase Resistance to Enterotoxigenic *Escherichia coli* Infection

**DOI:** 10.3390/microorganisms11010063

**Published:** 2022-12-25

**Authors:** Ayelen Baillo, Julio Villena, Leonardo Albarracín, Mikado Tomokiyo, Mariano Elean, Kohtaro Fukuyama, Sandra Quilodrán-Vega, Silvina Fadda, Haruki Kitazawa

**Affiliations:** 1 Laboratory of Technology, Reference Centre for Lactobacilli (CERELA-CONICET), Tucuman CP4000, Argentina; 2 Laboratory of Immunobiotechnology, Reference Centre for Lactobacilli (CERELA-CONICET), Tucuman CP4000, Argentina; 3 Food and Feed Immunology Group, Laboratory of Animal Food Function, Graduate School of Agricultural Science, Tohoku University, Sendai 981-8555, Japan; 4 Livestock Immunology Unit, International Education and Research Center for Food and Agricultural Immunology (CFAI), Graduate School of Agricultural Science, Tohoku University, Sendai 981-8555, Japan; 5 Laboratory of Food Microbiology, Faculty of Veterinary Sciences, University of Concepción, Chillán 3820572, Chile

**Keywords:** probiotics, lactic acid bacteria, *Lactiplantibacillus plantarum*, intestinal immune response, enterotoxigenic *Escherichia coli*

## Abstract

Currently, probiotic bacteria with not transferable antibiotic resistance represent a sustainable strategy for the treatment and prevention of enterotoxigenic *Escherichia coli* (ETEC) in farm animals. *Lactiplantibacillus plantarum* is among the most versatile species used in the food industry, either as starter cultures or probiotics. In the present work, the immunobiotic potential of *L. plantarum* CRL681 and CRL1506 was studied to evaluate their capability to improve the resistance to ETEC infection. In vitro studies using porcine intestinal epithelial (PIE) cells and in vivo experiments in mice were undertaken. Expression analysis indicated that both strains were able to trigger IL-6 and IL-8 expression in PIE cells in steady-state conditions. Furthermore, mice orally treated with these strains had significantly improved levels of IFN-γ and TNF-α in the intestine as well as enhanced activity of peritoneal macrophages. The ability of CRL681 and CRL1506 to beneficially modulate intestinal immunity was further evidenced in ETEC-challenge experiments. In vitro, the CRL1506 and CRL681 strains modulated the expression of inflammatory cytokines (IL-6) and chemokines (IL-8, CCL2, CXCL5 and CXCL9) in ETEC-stimulated PIE cells. In vivo experiments demonstrated the ability of both strains to beneficially regulate the immune response against this pathogen. Moreover, the oral treatment of mice with lactic acid bacteria (LAB) strains significantly reduced ETEC counts in jejunum and ileum and prevented the spread of the pathogen to the spleen and liver. Additionally, LAB treated-mice had improved levels of intestinal IL-10 both at steady state and after the challenge with ETEC. The protective effect against ETEC infection was not observed for the non-immunomodulatory TL2677 strain. Furthermore, the study showed that *L. plantarum* CRL1506 was more efficient than the CRL681 strain to modulate mucosal immunity highlighting the strain specific character of this probiotic activity. Our results suggest that the improved intestinal epithelial defenses and innate immunity induced by *L. plantarum* CRL1506 and CRL681 would increase the clearance of ETEC and at the same time, protect the host against detrimental inflammation. These constitute valuable features for future probiotic products able to improve the resistance to ETEC infection.

## 1. Introduction

Enterotoxigenic *Escherichia coli* (ETEC) is one of the most common causes of human diarrhea in developing countries [1] These bacteria can also affect farm animals. For example, it is the main bacterial etiologic agent of post-weaning diarrhea in pigs [2]. ETEC-associated disease causes huge economic losses in the global swine industry due to high morbidity and mortality, substantial veterinary costs, and stunted growth of animals [3]. Symptoms of ETEC infection include watery diarrhea with associated depression, loss of appetite, and dehydration. ETEC strains adhere to small intestinal epithelial cells (IECs) through flexible fimbriae present on their surface, which mediate recognition and adherence to the corresponding receptors [4]. ETECs expressing F4 fimbriae are the most prevalent strains in pigs [3]. After colonization, porcine ETEC strains produce one or more thermolabile (LT) and/or thermostable enterotoxins (ST) [2], which activate a flow of electrolytes towards the intestinal lumen, creating a hypertonic environment. Consequently, water moves from the epithelial cells into the intestinal lumen causing hypersecretory diarrhea. In addition to enterotoxins damage, the lipopolysaccharide (LPS) from the cell wall can induce intestinal damage through the stimulation of the inflammatory response [5]. The innate immune response of IECs is initiated when the pathogen-associated molecular pattern (PAMPs) such as LPS, binds to specialized pattern recognition receptors, including membrane-bound Toll-like receptors (TLRs). This interaction activates the signaling pathway for nuclear factor kappa B (NF-κB) and mitogen-activated protein kinase (MAPK) [6], leading to the transcriptional expression of various pro-inflammatory chemokines, cytokines, and antimicrobial peptides that trigger the recruitment and activation of inflammatory cells [7,8]. The TLR4-mediated production of inflammatory cytokines (TNF-α, IL-6, IL-17), chemokines chemoattractant for neutrophils (IL-8, CXCL5), monocytes (CCL2, CCL8) and lymphocytes (CXCL9, CXCL10, CXCL11) can contribute to intestinal tissue damage in pigs during ETEC infection if it is not regulated properly [5,9].

Currently, the treatment and prevention of ETEC in animals are based on the use of antimicrobials. This has led to the emergence of antibiotic resistant bacterial strains around the world [10]. Emerging clones carry multiple resistance genes, have high dissemination capacity and high pathogenicity [11]. Therefore, it is necessary to develop alternatives that help to reduce the impact of ETEC infection and eliminate the need for antimicrobial treatment. In this sense, probiotics represent an attractive strategy since they are selected from bacteria with not transferable antibiotic resistance, and they do not have the deleterious effect of antibiotics on the intestinal microbiota [12]. Research has clearly demonstrated the protective effects of probiotic microorganisms against pathogenic *E. coli*. Earlier studies performed with the probiotic strains *Bifidobacterium lactis* HN019 [13]. or *Lacticaseibacillus rhamnosus* HN001 [14]. demonstrated that their preventive administration to mice significantly improved the resistance against *E. coli* O157:H7 infection This effect was associated with an enhancement of intestinal immunity. Furthermore, by using a piglet model it was shown that *B. lactis* HN019 administration increased feed conversion efficiency during weaning and that this effect was associated with a reduction of the severity of weanling diarrhea [15]. A multispecies probiotic formulation was also evaluated in its capacity to diminish the severity of post-weaning diarrhea caused by ETEC on newly weaned pigs [16]. The multispecies probiotics improved growth performance by preserving the intestinal mucosa integrity and diminishing intestinal inflammatory factors like TNF-α. Similarly, the administration of *Pediococcus acidilactici* to weaned pigs was shown to increase resistance to ETEC challenge by modulating the expression of intestinal cytokines [17]. The probiotic treatment significantly reduced the attachment of ETEC to the intestinal mucosa in pigs and differentially regulated the expression of IL-6 and TNF-α. Our research group has experience identifying probiotic strains capable of beneficially regulating the intestinal immune system, increasing the resistance to ETEC infection [5,18,19]. These immunomodulatory probiotic strains, referred as immunobiotics, were selected by in vitro assays based on the porcine intestinal epitheliocyte cell line (PIE cells) developed by our group [20]. In previous studies, we have shown that TLR4 is strongly expressed in this cell line and that PIE cells can increase the expression of proinflammatory chemokines and cytokines in response to LPS stimulation [21]. In addition, by using this in vitro model we were able to select immunobiotic lactobacilli capable of modulating cytokines and chemokines expression caused by ETEC or LPS challenges [19,20].

*Lactiplantibacillus plantarum* can survive in a wide range of environmental niches including the gastrointestinal tract, easily colonizing the intestine of humans and other mammals [22,23]. In addition, many strains of this bacterial species have shown to possess beneficial properties for the host, including their ability to beneficially modulate the immune system [24]. Due to these properties, *L. plantarum* is considered one of the most widely used bacterial strains in the food industry both as a starter culture and as a probiotic [25]. Previously, we performed in vitro studies in PIE cells and in vivo studies in mice with different *L. plantarum* strains and we demonstrated that those lactic acid bacteria (LAB) possess a differential ability to modulate the respiratory and intestinal innate antiviral immune responses [26]. Of note, *L. plantarum* CRL1506 showed a remarkable capacity to beneficially regulate the mucosal antiviral immune response triggered by the activation of TLR3 [26,27,28]. The CRL1506 strain improves the production of type I interferons (IFNs) and antiviral factors and differentially regulates the expressions of inflammatory cytokines and chemokines in epithelial cells from the intestinal [26] and respiratory [28] tracts. Furthermore, in vivo studies in mice demonstrated that the oral treatment with *L. plantarum* CRL1506 can modulate the TLR3-mediated intestinal damage through its ability to reduce the expression of IL-15 in the intestinal epithelium and regulate the function of CD3^+^NK1.1^+^CD8αα^+^ intraepithelial lymphocytes [26,29]. Strains like *L. plantarum* TL2677 do not have those immunomodulatory capacities [26,30]. On the other hand, *L. plantarum* CRL681 has a proven technological potential as starter and bioprotective culture for meat and meat products. The CRL681 strain has remarkable acidogenic [31] and proteolytic [32,33,34] activities. In addition. *L. plantarum* CRL681 has bioprotective potential due to the high inhibitory activity toward *Escherichia coli* O157:H7 [35].

In the present work we aimed to deepen the characterization of the immunomodulatory properties of *L. plantarum* CRL1506 and CRL681 particularly focused on their ability to enhance intestinal immune responses and the resistance against ETEC. Therefore, we conducted in vitro studies in PIE cells to evaluate their capacity to modulate the innate immune response on the intestinal mucosa before and after ETEC challenge. In addition, we performed experiments in mice as preliminary studies to demonstrate in vivo the protective potential of CRL1506 and CRL681 strains against ETEC infection and to provide the scientific basis for carrying out future in vivo studies in pigs.

## 2. Materials and Methods

### 2.1. Bacterial Strains and Culture Conditions

*Lactiplantibacillus plantarum* CRL1506 was originally isolated from goat milk and CRL681 from fermented sausage. Both strains were obtained from the CERELA culture collection (Tucumán Argentina). *L. plantarum* TL2766, originally isolated from human feces, was included in the experiments as a non-immunomodulatory control strain. The TL2766 strain was obtained from the Meiji dairy culture collection (Tokyo, Japan).

For the experiments of this work, all *Lactiplantibacillus* strains were activated from frozen stock and grown on Mann-Rogosa Sharpe Agar (MRS Difco) at 37 °C. After 24 h of incubation, a single colony was transferred to MRS broth (MRS Difco) and was cultured at 37 °C for 24 h. Bacterial cells were then washed three times with phosphate-buffered saline (PBS) and adjusted to appropriate concentrations for in vitro and in vivo experiments using a microscope and a Petroff-Hausser counting chamber. They were stored at −80 °C until use [21].

Enterotoxigenic *Escherichia coli* (ETEC) strain 987P (O9: H-: 987 pilus +: heat stable toxin +) was obtained from the National Institute of Animal Health (Tsukuba, Japan) [21,36]. ETEC cells were cultured on blood agar (5% sheep blood) for 24 h at 37 °C, transferred to tryptic soy broth (TSB; Becton, Dickinson and Company, Franklin Lakes, NJ, USA) and cultured 20 h at 37 °C with shaking. After incubation, the bacterial subcultures were centrifuged at 5000× *g* for 10 min at 4 °C and washed with PBS (pH 7.2). Finally, the ETEC cells were suspended in Dulbecco’s Modified Eagle’s Medium (DMEM) for the experimental challenge with PIE cells. Live human ETEC O9, F4 pilus +, STp + kanamycin resistant strain was used for in vivo experiments as described below.

### 2.2. Porcine Intestinal Epitheliocyte Cells

PIE cells are untransformed intestinal cultured cells. They were originally derived from intestinal epithelium isolated from a non-suckling newborn pig [20]. PIE cells were maintained in DMEM (Invitrogen Corporation, Carlsbad, CA, USA) supplemented with 10% fetal bovine serum, 100 mg/mL penicillin and 100 U/mL streptomycin at 37 °C in a 5% CO_2_ atmosphere [21,36]. PIE cells were cultured in 250 mL flasks (1.0 × 10^6^ cells) for 5 days changing the culture medium every 1–2 days. After reaching 80–90% confluence, cells were subcultured in 24-well flasks for immunostimulation assays as described below.

### 2.3. Immunomodulatory Assay in PIE Cells

Twelve-well type I collagen coated plates (Iwaki, Tokyo, Japan) were used to seed the PIE cells (3 × 10^4^ cells/well) and they were cultured for 3 days. The medium was then replaced and the lactobacilli (10^8^ cells/mL) were added. They were shaken in a microplate mixer and co-cultured for 48 h at 37 °C in a 5% CO_2_ atmosphere. Each well was then vigorously washed with medium at least 3 times to remove bacteria. The gene expression of inflammatory cytokines (IL-6 and IL-8) and chemokines (CCL2, CCL8, CXCL5, CXCL9, CXCL10, and CXCL11), as well as negative regulators of TLR4 signaling (SIGIRR, Tollip, A20, Bcl-3, IRAK-M, and MKP-1), were studied without any inflammatory challenge (basal levels) or after a thermostable ETEC PAMPs challenge (5 × 10^7^ cells/mL) for 12 h using RT-PCR as described below.

### 2.4. Quantitative Expression Analysis by RT-PCR

We performed two-step real-time quantitative PCR to characterize the expression of selected genes in PIE cells as described before [13,32]. The primers used in this study were described previously [18,21,37]. The PCR cycling conditions were 2 min at 50 °C, followed by 2 min at 95 °C, and then 40 cycles of 15 s at 95 °C, 30 s at 60 °C, and 30 s at 72 °C. The reaction mixtures contained 5 μL of sample cDNA and 15 μL of master mix, including the sense and antisense primers. Expression of β-actin was used to normalize cDNA levels for differences in total cDNA levels in the samples. In ETEC challenge experiments, a relative index was calculated after normalization with β-actin and results were expressed as normalized fold expression based on challenged control PIE cells set as 1.0.

### 2.5. ETEC Challenge in Mice

This study was carried out in strict accordance with the recommendations of the Guide for the Care and Use of Laboratory Animals of the CERELA, Guide for Animal Experimentation. Five-week-old female BALB/c mice were obtained from the closed colony maintained at CERELA (Tucumán, Argentina). They were housed in plastic cages with controlled room temperature (22 ± 2 °C temperature, 55 ± 2% humidity) and mice were fed ad libitum with a conventional balanced diet. Researchers and personnel specialized in animal care and handling at CERELA ensured animal welfare. The health and behavior of the animals were monitored twice a day. The tests for each parameter studied were carried out in 5–6 mice per group. Animals were euthanized immediately after the time point was reached using xylazine and ketamine. No signs of discomfort or pain were observed and there were no deaths before the mice reached the end points.

*L. plantarum* CRL1506, CRL681 or TL2677 were administered orally to different groups of mice for 5 consecutive days at a dose of 10^8^ cells/mouse/day. On the sixth day, the lactobacilli-treated groups and the untreated control mice were orally inoculated with 200 mL of a bacterial suspension containing human ETEC O9, F4 pilus +, STp + kanamycin resistant strain (1 × 10^9^ cells) diluted with 0.1 M carbonate buffer (pH 9.0). Two days after ETEC inoculation, the mice were sacrificed to collect jejunum, ileum, spleen, and liver samples. The collected tissues were weighed and homogenized in BHI broth. Homogenates were plated on MAC agar plates containing kanamycin for ETEC counts. The results were expressed as logarithm of colony forming units (CFU) per gram of organ.

### 2.6. Ex Vivo Peritoneal Macrophage Phagocytosis Assay

Peritoneal macrophages were collected aseptically from mice as previously described [18,38]. Briefly, the inner skin was exposed, and cold PBS supplemented with 10% fetal calf serum by carefully injection into the peritoneal cavity. The liquid was collected, and the macrophages were washed twice with PBS containing bovine serum albumin (BSA). Cells were adjusted to a concentration of 1 × 10^6^ per ml. Phagocytosis was assessed using heat-treated *Saccharomyces boulardi*. For this purpose, mixtures of opsonized yeast in autologous mouse serum (10%) were added to 0.2 mL of macrophage suspension. Samples were incubated for 30 min at 37 °C. The percentage of phagocytosis was expressed as the percentage of phagocytic macrophages in 200 cells counted using a light microscope.

### 2.7. Bactericidal Activity of Peritoneal Macrophages

The bactericidal activity (oxidative blast) of peritoneal macrophages was measured using the nitro blue tetrazolium reduction test (NBT, Sigma-Aldrich, St. Louis, MO, USA) as previously described [18,38]. Briefly, peritoneal macrophages were obtained as described above and 200 µL of these cells were incubated with 120 µL of NBT reagent. Samples were incubated first at 37 °C for 10 min and then 10 min at room temperature. Then, NBT was added and incubated at 37 °C for 20 min. In the presence of oxidative metabolites, NBT (yellow) is reduced to formazan, which forms a blue precipitate. Finally, the samples were examined with a light microscope for blue precipitates. At random, 100 cells were counted and the percentage of NBT positive (+) cells was determined.

### 2.8. Cytokine Concentrations

Concentrations of cytokines were determined in blood and intestinal samples from lactobacilli-treated and control mice. Blood samples were obtained by cardiac puncture under anesthesia. Intestinal fluid samples were obtained as previously described (Indo et al., 2021). TNF-α, IL-6, IL-10, IFN-γ, chemokine KC (or CXCL1) and monocyte chemoattractant protein 1 (MCP-1) concentrations were measured with enzyme-linked immunosorbent assay (ELISA) kits following the manufacturer’s recommendations (R&D Systems, Minneapolis, MN, USA).

### 2.9. Statistical Analysis

Experiments were performed in triplicate and results expressed as the mean ± SD. For the comparison of two groups, the Student’s t-test was used after the verification of normal distribution. For the comparison of more than two groups, a one-way analysis of variance (ANOVA) was performed followed by and Tukey’s test. In all cases, a level of significance of *p* < 0.05 was considered.

## 3. Results

### 3.1. Effect of L. plantarum Strains on the Expression of Cytokines and the Negative Regulators of the TLR4 Signaling in PIE Cells

**Figure 1:**
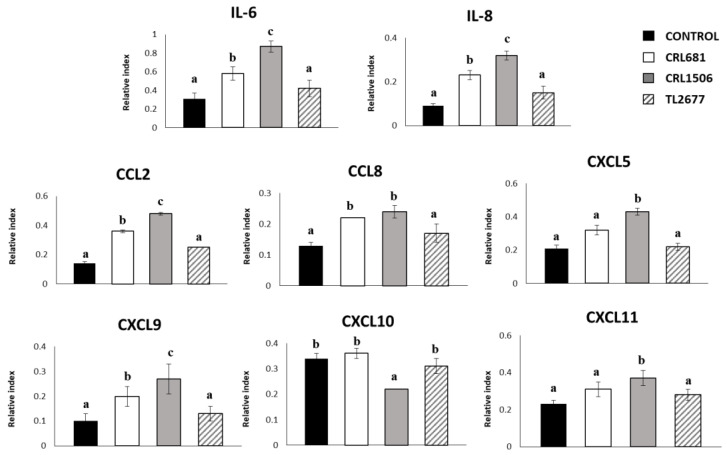
Effect of *Lactiplantibacillus plantarum* CRL681, CRL1506 or TL2677 on the expression of cytokines and chemokines in porcine intestinal epithelial (PIE) cells. PIE cells were stimulated with CRL681, CRL1506 or TL2677 strains for 48 h. Untreated cells were used as controls. The expression of cytokines (IL-6) and chemokines (CCL2, CXCL5, CXCL8, CXCL9, CXCL10, CXCL11, and CCL8) were studied at 48 h after lactobacilli stimulation (basal). The results represent three independent experiments. Results are expressed as mean ± SD. Bold letters indicate significant differences when compared to the control group (*p* < 0.05).

We evaluated whether *L. plantarum* CRL1506, CRL681 or TL2766 could modify the cytokine expression profile of PIE cells and whether the immunomodulatory property was strain specific. For this purpose, we comparatively analyzed the mRNA levels of IL-6 and the chemokines IL-8, CCL2, CCL8, CXCL5, CXCL9, CXCL10, and CXCL11 in PIE cells stimulated with these three bacterial strains (Figure 1). Stimulation of PIE cells with strains CRL1506 or CRL681 increased the expression of IL-6, IL-8, CCL2, CCL8 and CXCL9, while no significant differences were found in the levels of these inflammatory cytokines and chemokines between PIE cells treated with *L. plantarum* TL2677 and the control group. Only the strain CRL1506 was able to increase CXCL9 and CXCL11 levels compared to the control group, while CXCL10 levels decreased significantly in PIE cells treated with this *Lactiplantibacillus* strain. There was no significant difference in the levels of this chemokine among the other groups (Figure 1).

**Figure 2:**
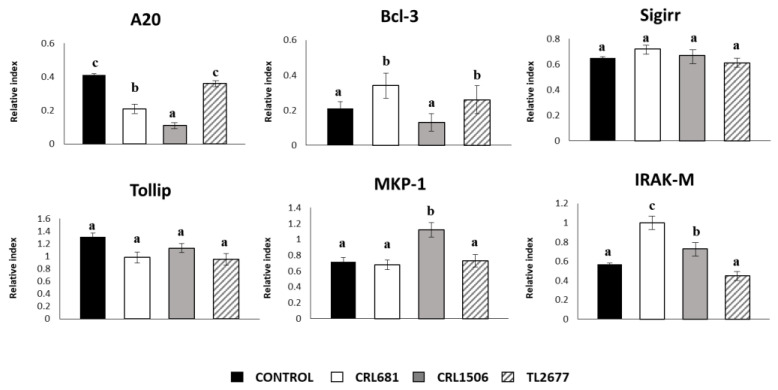
Effect of *Lactiplantibacillus plantarum* CRL681, CRL1506 or TL2677 on the expression of negative regulators of the Toll-like receptor (TLR) signaling pathway in porcine intestinal epithelial (PIE) cells. PIE cells were stimulated with CRL681, CRL1506 or TL2677 strains for 48 h. Untreated cells were used as controls. The expression of negative regulators of the TLR signaling pathway were studied at 48 h after lactobacilli stimulation (basal). The results represent three independent experiments. Results are expressed as mean ± SD. Bold letters indicate significant differences when compared to the control group (*p* < 0.05).

In addition, we evaluated the influence of the three *L. plantarum* strains on the expression of the negative regulators of the TLR4 signaling in PIE cells (Figure 2). No significant differences were observed in the expression of SIGIRR and Tollip when untreated control PIE cells and those treated with the different *L. plantarum* strains were compared. In addition, *L. plantarum* CRL681 and CRL1506 were able to reduce the expression of A20 in PIE cells and both strains increased the expression of Bcl-3 and IRAK-M. *L. plantarum* CRL1506 was the only strain capable of increasing the expression of MKP-1 compared to the control group (Figure 2).

### 3.2. Effect of L. plantarum Strains on ETEC-Activated Innate Immune Response in PIE Cells

**Figure 3:**
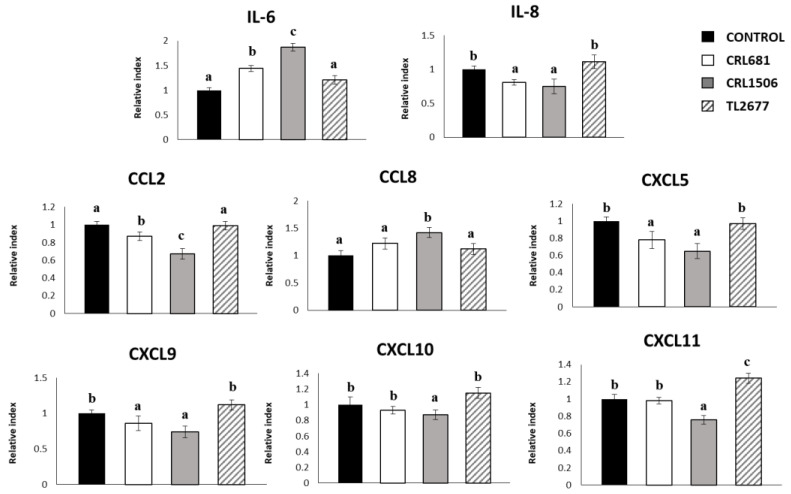
Effect of *Lactiplantibacillus plantarum* CRL681, CRL1506 or TL2677 on the expression of cytokines and chemokines in porcine intestinal epithelial (PIE) cells after Enterotoxigenic *Escherichia coli* (ETEC) challenge. PIE cells were pre-treated with CRL681, CRL1506 or TL2677strains for 48 h and then stimulated with heat-stable ETEC pathogen-associated molecular patterns (PAMPs). Non-lactobacilli treated cells were used as controls. The expression of cytokines (IL-6) and chemokines (CCL2, CXCL5, CXCL8, CXCL9, CXCL10, CXCL11, and CCL8) were studied at 12 h after heat-stable ETEC PAMPs challenge. The results represent three independent experiments. Results are expressed as mean ± SD. Bold letters indicate significant differences when compared to the control group (*p* < 0.05).

To study modulation of cytokines and chemokines in the context of inflammation, PIE cells were treated with *L. plantarum* CRL681, CRL1506 or TL2677. Then, cells were challenged with thermostable ETEC PAMPs. These molecular patterns can trigger TLR4 activation in this cell line, as we described previously [18,36]. Non-*Lactiplantibacillus* treated PIE cells challenged with ETEC were used as controls. The ETEC PAMPs significantly increased the expression of all inflammatory cytokines and chemokines in all experimental groups as shown in Figure 3, when compared to basal levels. The mRNA expression levels of IL-6 were significantly higher in cells treated with CRL681 and CRL1506 strains. On the other hand, the expressions of IL-8, CCL2, CXCL5 and CXCL9 were lower in PIE cells treated with these strains compared to controls. Interestingly, only *L. plantarum* CRL1506 enhanced the expression of CCL8 (Figure 3). *L. plantarum* TL2677 increased the expression of CXCL11 after exposure to ETEC, while strain CRL1506 reduced the expression of this chemokine (Figure 3). No significant differences were observed in the levels of CXCL10 between the groups treated with lactobacilli and the control group after ETEC challenge (Figure 3).

**Figure 4:**
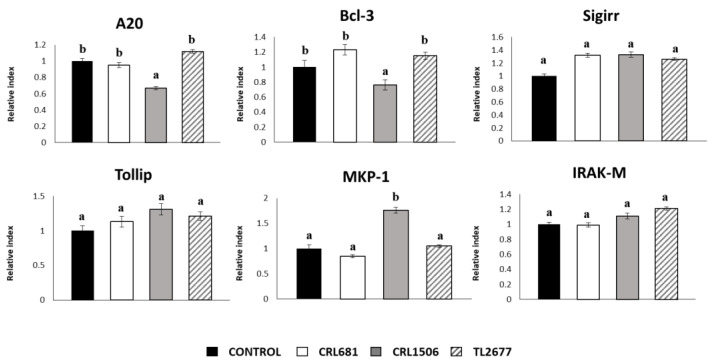
Effect of *Lactiplantibacillus plantarum* CRL681, CRL1506 or TL2677 on the expression of negative regulators of the Toll-like receptor (TLR) signaling pathway in porcine intestinal epithelial (PIE) cells after Enterotoxigenic *Escherichia coli* (ETEC) challenge. PIE cells were pre-treated with CRL681, CRL1506 or TL2677 strains for 48 h and then stimulated with heat-stable ETEC pathogen-associated molecular patterns (PAMPs). Non-lactobacilli treated cells were used as controls. The expression of negative regulators of the TLR signaling pathway were studied at 12 h after heat-stable ETEC PAMPs challenge. The results represent three independent experiments. Results are expressed as mean ± SD. Bold letters indicate significant differences when compared to the control group (*p* < 0.05).

When the negative regulators of the TLR signaling pathway were investigated after exposure to ETEC PAMPs, it was found that only *L. plantarum* CRL1506 reduced the expressions of A20 and Bcl-3 (Figure 4). This strain also increased the levels of MKP-1 in ETEC-challenged PIE cells. No significant differences were observed in the levels of the other mediators analyzed in this study (Figure 4).

### 3.3. L. plantarum CRL681 and CRL1506 Modulate Intestinal Immunity In Vivo

**Figure 5:**
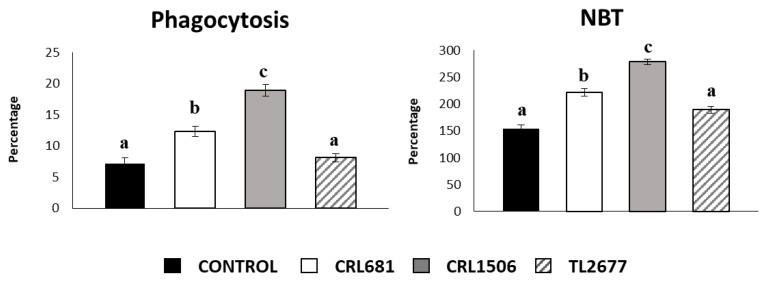
Effect of *Lactiplantibacillus plantarum* CRL681, CRL1506 or TL2677 on peritoneal macrophages activities. Mice were orally treated with *L. plantarum* CRL681, CRL1506 or TL2677 (10^8^ cells/mouse per day for 5 consecutive days). Untreated mice were used as controls. One day after the last *lactobacilli* administration, phagocytic and bactericidal (oxidative burst) activities of peritoneal macrophages were determined. The results represent three independent experiments. Results are expressed as mean ± SD. Bold letters indicate significant differences when compared to the control group (*p* < 0.05).

In addition, the ability of the different *L. plantarum* strains to stimulate macrophages was evaluated. For this purpose, ex vivo analysis of the phagocytic and bactericidal activity of the peritoneal macrophages were carried out. *L. plantarum* strains CRL681 and CRL1506 significantly increased the phagocytic activity of peritoneal macrophages, while this effect was absent in the strain TL2677 (Figure 5). In addition, a significant difference in the percentage of phagocytosis between the strains CRL681 and CRL1506 was observed, the latter strain showing higher phagocytic activity (Figure 5). To study the activation of the respiratory burst in peritoneal macrophages, we used the NBT method as previously described [18]. The treatment with the CRL1506 strain was more effective to enhance the percentage of NBT^+^ cells in the macrophage population obtained from the peritoneal cavity than the treatment with the CRL681 strain (Figure 5). Of note, *L. plantarum* TL2677 did not induce significant changes compared to the control group (Figure 5).

**Figure 6:**
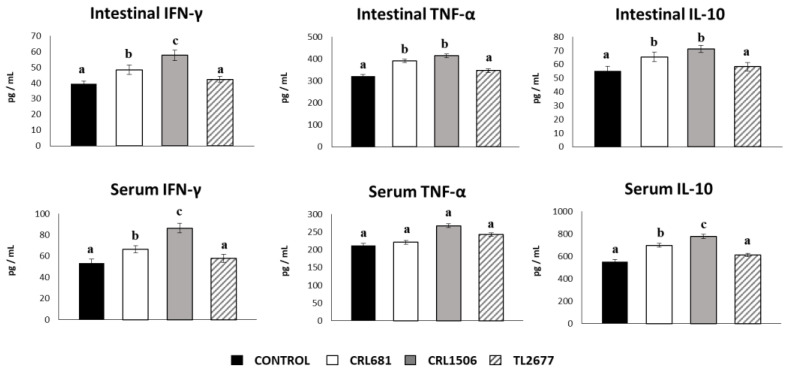
Effect of *Lactiplantibacillus plantarum* CRL681, CRL1506 or TL2677 on intestinal and serum cytokines of adult immunocompetent mice after enterotoxigenic *Escherichia coli* (ETEC) challenge. Mice were orally treated with *L. plantarum* CRL681, CRL1506 or TL2677 (10^8^ cells/mouse per day for 5 consecutive days) and then challenged orally with ETEC F4 strain (10^9^ cells). Mice with no *lactobacilli* treatment and challenged with ETEC were used as controls. Two days after the challenge, the concentrations of tumor necrosis factor (TNF)-α, interferon (IFN)-γ, and interleukin (IL)-10 in intestinal fluid and serum were determined by ELISA. The results represent three independent experiments. Results are expressed as mean ± SD. Bold letters indicate significant differences when compared to the control group (*p* < 0.05).

We also analyzed the cytokine concentrations in the intestinal fluid and serum obtained from mice treated with lactobacilli to determine the local and systemic effects induced by the *L. plantarum* strains (Figure 6). Both *L. plantarum* CRL681 and CRL1506 increased the levels of intestinal and serum IFN-γ. The concentrations of this cytokine found in the group stimulated with CRL1506 was higher than the CRL681 group (Figure 6). In addition, *L. plantarum* CRL681 and CRL1506 increased the level of intestinal TNF-α, while no differences were observed for serum TNF-α between the groups. Increased levels of IL-10 were found in both the intestinal fluid and serum of CRL1506- and CRL681-treated mice. However, serum levels of this immunoregulatory cytokine were significantly higher in mice treated with *L. plantarum* CRL1506 compared to those that received the CRL681 strain (Figure 6). No significant differences were observed between mice treated with *L. plantarum* TL2677 and control animals when the concentrations of intestinal and serum cytokines were analyzed (Figure 6).

### 3.4. L. plantarum CRL681 and CRL1506 prevent the Spread of ETEC and Modulate the Expression of Intestinal Cytokines after Bacterial Challenge

**Figure 7:**
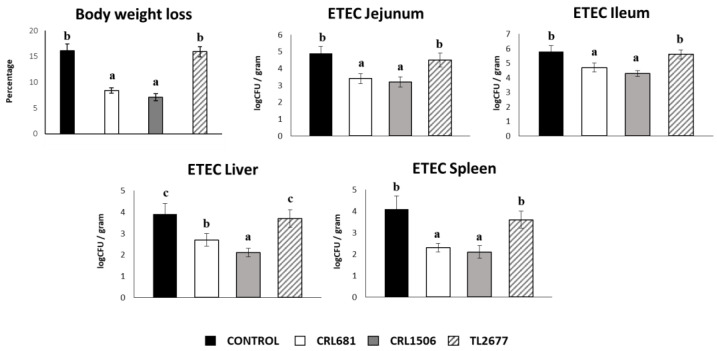
Immunomodulatory effect of *Lactiplantibacillus plantarum* CRL681, CRL1506 or TL2677 in mice in response to enterotoxigenic *Escherichia coli* (ETEC) challenge. Mice were orally treated with *L. plantarum* CRL681, CRL1506 or TL2677 (10^8^ cells/mouse per day for 5 consecutive days) and then challenged orally with ETEC F4 strain (10^9^ cells). Mice with no lactobacilli treatment and challenged with ETEC were used as controls. ETEC counts in jejunum, ileum, liver, and spleen were determined two days after the challenge. Values are means ± SD. Bold letters indicate significant differences when compared to the ETEC control group (*p* < 0.05).

In order to evaluate the effect of *L. plantarum* strains on resistance to ETEC infection, body weight loss and bacterial counts in the jejunum, ileum, liver and spleen of infected mice were determined two days after the challenge. We evaluated body weight loss to study the general health state of mice (Figure 7). The infection with ETEC significantly increased the body weight loss of mice we described previously [37]. Of note, mice treated with the CRL1506 and CRL681 strains had significantly lower percentages of body weight loss than controls. *L. plantarum* CRL681 and CRL1506 were also able to significantly reduce ETEC counts in jejunum and ileum compared to controls (Figure 7). Furthermore, both lactobacilli treatments prevented the spread of the pathogen to the spleen and liver. There were no significant differences in body weight loss and the pathogen’s counts observed in the different organs of the mice treated with TL2677 and the control group (Figure 7).

**Figure 8:**
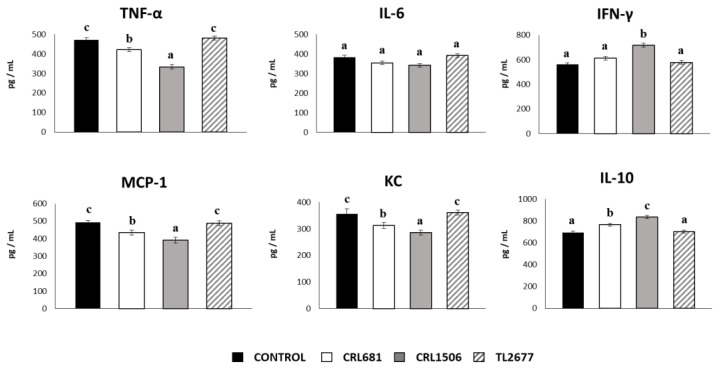
Immunomodulatory effect of *Lactiplantibacillus plantarum* CRL681, CRL1506 or TL2677 in mice in response to enterotoxigenic *Escherichia coli* (ETEC) challenge. Mice were orally treated with *L. plantarum* CRL681, CRL1506 or TL2677 (10^8^ cells/mouse per day for 5 consecutive days) and then challenged orally with ETEC F4 strain (10^9^ cells). Mice with no *lactobacilli* treatment and challenged with ETEC were used as controls. The intestinal levels of tumor necrosis factor (TNF)-α, interferon (IFN)-γ, interleukin (IL)-6, IL-10, chemokine KC (or CXCL1), and monocyte chemoattractant protein 1 (MCP-1) were determined two days after the challenge with ETEC. Values are means ± SD. Bold letters indicate significant differences when compared to the ETEC control group (*p* < 0.05).

The levels of TNF-α, IL-6, IFN-γ, MCP-1, KC and IL-10 were also quantified in the gut mucosa of mice treated with lactobacilli and challenged with ETEC. Both *L. plantarum* CRL681 and CRL1506 significantly reduced the intestinal levels of TNF-α, KC and MCP-1 in comparison with the controls, being the CRL1506 strain the most effective to induce the decrease of these cytokines (Figure 8). Mice treated with strains CRL681 or CRL1506 showed higher intestinal IL-10 concentrations than controls, while only CRL1506 increased IFN-γ levels compared to the control group (Figure 8). No significant differences were observed in IL-6 levels between the studied groups. In addition, no differences were detected in intestinal cytokines levels between the group treated with *L. plantarum* TL2677 and the control group (Figure 8).

## 4. Discussion

LAB of various species can be used as probiotics for animals with the aim to control pathogenic microorganisms and improve natural defense mechanisms, reducing health problems and therefore increasing productivity [39,40]. *L. plantarum* is among the most versatile species used for decades in the food industry either as starter cultures or probiotics [25]. They are applied as starter cultures to produce cheeses, sausages, olives and a wide variety of fermented foods and beverages, contributing to their organoleptic properties, flavor and texture. This outstanding versatility and metabolic activity can be explained by its big genome (2.91 to 3.7 Mbp in length) compared to other LAB such as *Latilactobacillus curvatus* or *Lactobacillus sakei* which account for less than 1.9 Mbp [41,42] This feature contributes to its survival capability in a wide range of environmental niches including plants, fermented foods, and the gastrointestinal tract humans and other mammals [22,23]. The increasing significance as probiotic of this species are mainly linked to health promotion in humans and animals [24,43,44,45,46,47,48,49]. However, their beneficial effects are strain dependent and not universal. Therefore, the probiotic properties need to be characterized on a strain level.

In this context, the immunobiotic potential of two *L. plantarum* strains with different origins and probiotic/technological properties was studied and compared to evaluate their capability to improve the resistance to ETEC infection. *L. plantarum* CRL681, originally isolated from fermented sausages, has an efficient acidogenic activity that guarantees safety and texture development during ripening [31,44]. In addition, detailed peptidomic studies confirmed its peptidogenic ability and its capacity to increase free amino acid contents in meat or fermented-meat models [31,32,33]. The CRL681 strain is capable of degrading biogenic amines in vitro and lacks the ability to produce them [40]. Moreover, this strain has remarkable bioprotective potential due to the high inhibitory activity toward *E. coli* O157:H7 [35]. On the other hand, *L. plantarum* CRL1506, originally isolated from goat milk, has demonstrated to possess remarkable immunomodulatory activities in the context of antiviral immunity [26,27,28,29,46], although its capacity to modulate antibacterial immune responses in the intestinal tract has not been explored in depth.

In the present study, we demonstrated that both CRL681 and CRL1506 strains can modulate the intestinal immune response in steady-state conditions. *L. plantarum* CRL681 and CRL1506 triggered the expression of IL-6 and IL-8 in porcine IECs. Furthermore, mice orally treated with these strains had significantly improved levels of IFN-γ and TNF-α in the intestine as well as enhanced activity of peritoneal macrophages. Studies evaluating the effect of lactobacilli strains with the capacity to reduce the severity of intestinal infections found that the most remarkable effect was the increase in the intestinal levels of TNF-α, IFN-γ, IL-1β, IL-6, and IL-12 for the mice treated with the probiotic strains, as well as the phagocytic activity of intestinal and peritoneal macrophages [47,48]. In vitro and ex vivo studies in a primary culture of IECs demonstrated that probiotic lactobacilli interact with these cells and induce release of IL-6 [47,49]. This cytokine was shown to regulate the survival and proliferation of IECs as well as to stimulate immune cells in the intestinal mucosa [50]. Autophagy in IECs is involved in the homeostatic control of cell death and differentiation. It was shown that IECs have a high basal level of autophagy that is regulated by TLR-mediated IL-8 production [51]. On the other hand, probiotic bacteria can stimulate macrophages by increasing their phagocytic activities and their capacity to produce cytokines like IFN-γ and TNF-α. This macrophage activity is essential for the protection against infections [52]. Thus, our results suggest that both CRL681 and CRL1506 strains have the capacity to interact with IECs and macrophages in the gut, reinforcing the epithelial defenses and stimulating immune responses. In fact, the ability of *L. plantarum* CRL681 and CRL1506 to beneficially modulate intestinal immunity was put into greater evidence in the experiments in which challenges with ETEC were performed.

In this work, using the in vitro PIE cell system we observed that *L. plantarum* CRL681 and CRL1506 differentially modulated the innate immune responses of porcine IECs triggered by ETEC challenge. The CRL1506 and CRL681 strains modulated the expression of inflammatory cytokines (IL-6) and chemokines (IL-8, CCL2, CXCL5 and CXCL9) in ETEC-stimulated PIE cells. Furthermore, our studies in the mice model of ETEC infection demonstrated in vivo the ability of *L. plantarum* CRL681 and CRL1506 to beneficially regulate the immune response against this pathogen. In our hands, the oral treatment of mice with CRL681 or CRL1506 strains significantly reduced body weight loss as well as ETEC counts in jejunum and ileum and prevented the spread of the pathogen to the spleen and liver. This protective effect could be related to a differential regulation of cytokines and chemokines in the intestinal mucosa, particularly in IECs. Our results are in line with previous works demonstrating that probiotic microorganisms can regulate the immune response against ETEC in both mice models [13,14] and pigs [15,16,17] as well as in IECs [53,54]. *Lacticaseibacillus rhamnosus* GG and *Bifidobacterium animalis* MB5 protected human Caco-2 cells from the ETEC K88-associated inflammation by reducing IL-1β and TNF-α and enhancing TGF-β1 expression [53,55]. *Enterococcus faecium* HDRsEf1 has been shown to have positive effects on piglet diarrhea, and studies performed in the porcine IPEC-J2 cell line demonstrated that this effect is partially related to its ability to reduce the expression of IL-8 after ETEC challenge, protecting IECs from the acute inflammatory response [54]. In addition, it was shown that the porcine IECs line IPEC-1 treated with *L. plantarum* CGMCC 1258 had reduced expressions of IL-1α, IL-6, IL-8 and TNF-α after the challenge with ETEC K88. This effect was associated with the ability of the CGMCC 1258 strain to regulate MAPK and NF-κB signaling pathways [56]. Our previous studies in PIE cells showed a reduction in the activation of NF-κB and MAPK signaling pathways and in the expression of some inflammatory cytokines and chemokines in ETEC-challenged PIE cells, preventively stimulated with *Lactobacillus jensenii* TL2937 [21], or *Bifidobacterium breve* M-16V [36].

Then, our results suggest that the improved intestinal epithelial defenses and innate immunity induced by *L. plantarum* CRL681 and CRL1506 would increase the clearance of ETEC and at the same time, protect the host against detrimental inflammation. The activation of TLR4 in the intestinal mucosa induce the production of cytokines to stimulate the recruitment and activation of inflammatory cells. Although this mechanism is a key primary line of host defense, prolonged or dysregulated proinflammatory response may lead to tissue damage and dysfunction [57]. Thus, the reduction of the intestinal levels of TNF-α, MCP-1 and KC induced by the CRL681 and CRL1506 strains could indicate a better control of inflammation and its detrimental effects. Furthermore, it was observed that mice treated with *L. plantarum* CRL681 or CRL1506 had improved levels of intestinal IL-10 both at steady state and after the challenge with ETEC. Studies performed in healthy adult volunteers challenged with ETEC demonstrated that higher pre-challenge concentrations of IL-10 were associated with protection from ETEC diarrhea [9]. In addition, in agreement with our results it was reported that the treatment of mice with *L. plantarum* CCFM1143 was able to alleviate diarrhea caused by ETEC infection, and that this beneficial effect was associated with reductions of TNF-α and improvements of IL-10 [58]. Furthermore, in line with the relevant role of IL-10 in controlling TNF-α production, our studies showed that in baseline determinations the levels of this regulatory cytokine increased only 1.4 times in animals treated with CRL1506 or CRL681 strains compared to controls and was not able to decrease the levels of the inflammatory cytokine. In contrast, in the ETEC-challenge experiments, IL-10 was augmented more than 10-fold compared to baseline values and 1.6-fold between CRL1506 and CRL681 versus controls.

We have previously demonstrated that the beneficial effects of immunobiotic bacteria in the context of TLR4-triggered inflammation are mediated by a differential modulation of negative regulators of the TLR signaling pathway [21,36]. Then, we also evaluated here the ability of *L. plantarum* CRL681 or CRL1506 to modulate the expression of SIGIRR, Tollip, A20, Bcl-3, IRAK-M, and MKP-1 in porcine IECs. We found that both strains upregulated the expression of IRAK-M and reduced A20 in PIE cells without ETEC challenge. In addition, CRL1506 and CRL681 increased the expressions of MKP-1 and Bcl-3, respectively. The TLR negative regulators play important roles in the maintenance of intestinal hemostasis as well as in the control of immune responses against pathogens. IRAK-M exerts its regulatory effect by acting on the TRAF6/IRAK-1 complex, diminishing the activation of NF-κB and MAPK pathways [59]. It was described that LPS challenge induce the expression of IRAK-M, and that the tolerance to TLR4 activation is diminished in IRAK-M-deficient cells [60]. On the other hand, it was reported that A20 [61,62] and Bcl-3 [63] regulate TLR4 signaling pathway by inducing the inhibition of NF-κB activation while MKP-1 inactivates the MAPK p38 signaling pathway [64]. Thus, it is tempting to speculate that lactobacilli would induce a differential expression of negative regulators of the TLR4 pathway in the intestinal epithelium, and when the challenged with ETEC is induced, this signaling pathway would be differentially regulated allowing an efficient induction of an antibacterial state in the gut, and the protection against the inflammatory-mediated damage.

Of note, the immunostimulatory activity of the CRL1506 and CRL681 strains were not achieved by *L. plantarum* TL2677 in porcine IECs. In addition, the protective effect of *L. plantarum* CRL681 and CRL1506 against ETEC infection was not observed for the TL2677 strain in mice, highlighting that the immunomodulatory effects are strain specific. Furthermore, the analysis of the immunomodulatory activities in steady state conditions as well as the immune responses triggered by ETEC challenge showed that *L. plantarum* CRL1506 is more efficient than CRL681 strain to modulate mucosal immunity. In line with our results, it was shown that different strains had individual capacities to regulate TLR4-NF-κB signaling pathway and the expression of IL-8 in HEK cells as well as to regulate trans-epithelial electrical resistance and tight junction integrity in LPS-challenged Caco-2 monolayers [65]. The different abilities of the *L. plantarum* strains studied here to influence the immune response against ETEC could be associated with their distinct capacity to modulate negative regulators of TLR. While the TL2677 did not modulate the expression of TLR negative regulators, *L. plantarum* CRL1506 was more efficient than the CRL681 strain to upregulate IRAK-M expression and diminish A20. Moreover, when the TLR negative regulators were evaluated in PIE cells challenged with ETEC, only cells treated with *L. plantarum* CRL1506 showed differences in the expressions of A20, Bcl-3 and MKP-1. Interestingly, despite the more notable changes in the expression of immune factors both in vitro and in vivo induced by the CRL1506 strain, no significant differences in ETEC counts were observed when compared to mice treated with *L. plantarum* CRL681. This may be due to the fact that in this work only one post-infection point was studied. Perhaps a study of the kinetics of ETEC clearance could find differences between the two treatments. In addition, a further study to evaluate whether a combination of both *L. plantarum* CRL1506 and CRL681 could improve immunity more effectively than individual strains and induce a more efficient clearance of ETEC in mice intestine would be of value. Another important point for future studies is to find the bacterial molecules and immune receptors involved in the induction of TLR negative regulators in the intestinal epithelium by *L. plantarum*, which also explain the differences between the strains. Our recent comparative genomic study showed a great variability in the predicted surface proteins of *L. plantarum* strains, including CRL1506 and CRL681 [30]. These results suggest that the surface molecules could be involved in their differential ability to modulate the intestinal innate immune response against ETEC.

## 5. Conclusions

Our results demonstrated that the improved intestinal epithelial defenses and innate immunity induced by *L. plantarum* CRL1506 and CRL681 would increase the clearance of ETEC and at the same time, the differential expression of negative regulators of the TLR4 pathway in the intestinal epithelium allowing the establishment of an antibacterial state in the gut, and the protection against the inflammatory-mediated damage. These findings constitute valuable features for a future probiotic culture for animal feed able to improve the resistance to ETEC infection. The preliminary studies carried out in this work using porcine IECs and mice provide the scientific basis for carrying out in vivo studies in pigs to reliably demonstrate the protective potential of CRL1506 and CRL681 strains against ETEC infection in the porcine host.

## Data Availability

Data are contained within this article.
